# Development of a Small-Sized, Flexible, and Insertable Fiber-Optic Radiation Sensor for Gamma-Ray Spectroscopy

**DOI:** 10.3390/s150921265

**Published:** 2015-08-28

**Authors:** Wook Jae Yoo, Sang Hun Shin, Dong Eun Lee, Kyoung Won Jang, Seunghyun Cho, Bongsoo Lee

**Affiliations:** 1 School of Biomedical Engineering, College of Biomedical & Health Science, BK21 Plus Research Institute of Biomedical Engineering, Konkuk University, 268 Chungwon-daero, Chungju-si, Chungcheongbuk-do 380-701, Korea; E-Mails: wonzip@kku.ac.kr (W.J.Y.); shshin9431@gmail.com (S.H.S.); lde4757@naver.com (D.E.L.); kko988@kku.ac.kr (K.W.J.); 2 Department of Organic Materials & Fiber Engineering, College of Engineering, Soongsil University, 369 Sangdo-ro, Dongjak-gu, Seoul-si 156-743, Korea; E-Mail: scho@ssu.ac.kr

**Keywords:** gamma-ray spectroscopy, fiber-optic radiation sensor, multichannel analyzer, plastic optical fiber, LYSO:Ce

## Abstract

We fabricated a small-sized, flexible, and insertable fiber-optic radiation sensor (FORS) that is composed of a sensing probe, a plastic optical fiber (POF), a photomultiplier tube (PMT)-amplifier system, and a multichannel analyzer (MCA) to obtain the energy spectra of radioactive isotopes. As an inorganic scintillator for gamma-ray spectroscopy, a cerium-doped lutetium yttrium orthosilicate (LYSO:Ce) crystal was used and two solid-disc type radioactive isotopes with the same dimensions, cesium-137 (Cs-137) and cobalt-60 (Co-60), were used as gamma-ray emitters. We first determined the length of the LYSO:Ce crystal considering the absorption of charged particle energy and measured the gamma-ray energy spectra using the FORS. The experimental results demonstrated that the proposed FORS can be used to discriminate species of radioactive isotopes by measuring their inherent energy spectra, even when gamma-ray emitters are mixed. The relationship between the measured photon counts of the FORS and the radioactivity of Cs-137 was subsequently obtained. The amount of scintillating light generated from the FORS increased by increasing the radioactivity of Cs-137. Finally, the performance of the fabricated FORS according to the length and diameter of the POF was also evaluated. Based on the results of this study, it is anticipated that a novel FORS can be developed to accurately measure the gamma-ray energy spectrum in inaccessible locations such as narrow areas and holes.

## 1. Introduction

Gamma-rays emitted from radioactive sources have specific energies. Due to the fact that gamma-rays are uncharged and indirectly ionizing radiation, gamma detection depends on causing the gamma-ray photons to undergo interactions in the absorbing material. When gamma-rays interact with a scintillator, they produce charged electrons mainly by three different interactions, a photoelectric effect, Compton scattering, and pair production. These electrons give rise to scintillating light, making it is possible to analyze and compare radioactive isotopes by measuring the total energy absorbed per interaction (*i.e*., pulse height) according to the specific energies of gamma-rays [1].

For radiation detection or gamma-ray energy spectroscopy, a scintillation detector using a thallium-activated sodium iodide (NaI:Tl) crystal is traditionally used in nuclear medicine and environmental measurements. Although an NaI:Tl crystal-based energy spectrometer has high efficiency and adequate energy resolution, it requires a large sensing volume for gamma-ray energy spectroscopy because the density and the effective atomic number (*i.e*., Z-number) of the NaI:Tl crystal are relatively lower than those of other inorganic scintillator crystals, such as bismuth germinate (BGO), cerium-doped gadolinium oxyorthosilicate (GSO:Ce), cerium-doped yttrium orthosilicate (YSO:Ce), and cerium-doped lutetium yttrium orthosilicate (LYSO:Ce) [2]. Its large sensing volume and lack of flexibility prohibit the detection of gamma-rays under challenging geometrical conditions. Furthermore, the NaI:Tl crystal must be sealed within an air-tight container, as it has a hygroscopic characteristic [1,2,3,4]. To overcome these problems and also allow real-time and remote measurements in harsh environments, a newly designed gamma-ray spectrometer with small size, flexibility, and non-hygroscopicity is required.

Radiation sensors using an optical fiber have been developed in conjunction with many kinds of organic or inorganic scintillators; most of them, however, can only measure scintillating light intensity [5,6,7]. Although existing fiber-optic radiation sensors (FORSs) have many advantages, such as small sensing volume, high spatial resolution, good flexibility, real-time sensing, remote operation, and immunity to high electromagnetic interference (EMI) [8,9,10], they have not been used for accurate discrimination of radioactive isotopes using a spectroscopic technique [11,12,13,14]. In previous reports, for radiation energy measurements, an optical fiber was only used to transmit a digital signal from an NaI:Tl scintillation detector module as a fiber-optic data link [15]. A scintillation counter that uses a Perspex light guide with a diameter of 5.08 cm, a plastic scintillator with 5.08 cm diameter and 2.54 cm thickness, and a pulse height analyzer was also introduced [16]. However, very thick and rigid Perspex with reflective wrapping was used as a light guide instead of an optical fiber and, accordingly, the total internal reflection ratio and the transmission rate of the scintillating light were low. Consequently, it could obtain the pulse height according to the length of the light guide but could not obtain the accurate gamma-ray energy spectrum.

Recently, a feasibility experiment on a novel FORS for gamma-ray spectroscopy was carried out by our research team [17]. In this experiment, the scintillating light generated from three types of inorganic scintillators, BGO, YSO:Ce, and LYSO:Ce, was measured and we selected LYSO:Ce crystal as an adequate scintillator because it provided the highest scintillating light output. Also, the gamma-ray energy spectra for sodium-22 (Na-22), cesium-137 (Cs-137), and cobalt-60 (Co-60) were measured successfully by using the FORS. However, FORS for gamma-ray spectroscopy have still not been deployed on a commercial scale due to both their small sensing volume, which cannot completely absorb the charged particle energy, and the light attenuation rate in the receiving optical fiber. 

The main purpose of the present study is to demonstrate that the proposed FORS can discriminate different gamma-ray emitters by measuring their inherent energy spectra, even when radioactive isotopes are mixed. For remote sensing and gamma-ray energy spectroscopy, we fabricated a small, flexible, and insertable FORS using a sensing probe, a plastic optical fiber (POF), a photomultiplier tube (PMT)-amplifier system, and a multichannel analyzer (MCA). By using the POF to transmit scintillating light with the total internal reflection, the FORS can be applied to measure various radioactive isotopes and radioactive contamination in inaccessible locations, such as narrow areas, pipes, and holes. In the proposed sensor, a POF is used to guide scintillating light generated from an inorganic scintillator crystal in the sensing probe to a PMT. A PMT-amplifier system is used to convert light signals to electrical signals and the amplified voltage signals are measured by an MCA. In this study, we determined the length of the inorganic scintillator crystal to obtain the gamma-ray energy spectrum of radioactive isotopes considering the absorption efficiency and evaluated the performance of the fabricated FORS according to the length and diameter of the POF. First, we measured the energy spectra of radioactive isotopes using the scintillating light transmitted via the POF and compared the results with the gamma-ray energy spectra obtained using a conventional high-purity germanium (HPGe) detector. The response of the proposed FORS according to the radioactivity was then obtained. In addition, we demonstrated that the species of radioactive isotopes can be discriminated using the measured gamma-ray energy spectra. 

## 2. Materials and Experimental Setup

As a sensing element of the FORS probe, an LYSO:Ce crystal (LYSO:Ce, Advanced Microwave Technologies Solution Co., Ltd., Seoul, Korea) was used for gamma-ray energy spectroscopy. Table 1 shows the physical properties of the LYSO:Ce inorganic scintillator crystal used in this study. The colorless and transparent LYSO:Ce crystal has many advantages including low cost, high density, high effective Z-number, high light output, fast decay time, and good energy resolution. In addition, it has a non-hygroscopic characteristic and high sensitivity against gamma-rays. The light yield of the LYSO:Ce crystal is 85% relative to NaI:Tl. However, LYSO:Ce has about two and three times more light output compared to GSO and BGO, respectively. The peak emission wavelength and the decay time of an LYSO:Ce crystal are about 402 nm and 40 ns, respectively.

In order to transmit scintillating light with the total internal reflection, a step-index multimode fiber (CK-120, Mitsubishi Rayon Co., Ltd., Tokyo, Japan) was chosen as the POF in this study. Diameters are 2.95 ± 0.18 mm for the core only and 3.00 ± 0.18 mm including the cladding. The materials of the core and the cladding are polymethylmethacrylate (PMMA) and fluorinated polymer, respectively. Accordingly, the refractive indices of the core and the cladding are 1.49 and 1.402, respectively, and the numerical aperture (NA) is approximately 0.5. The maximum transmission loss of the POF is 200 dB/km when used with 650 nm collimated light. In a previous report, no significant degradation in the attenuation of POF was observed for irradiation up to 3.5 kGy [18].

**Table 1:** Physical properties of LYSO:Ce crystal.

Density (g/cm^3^)	Melting Point (°C)	Refractive Index	Decay Time (ns)	Peak Emission (nm)	Light Yield (%) (Relative to Nal:Tl)
7.40	2050	1.82	40	402	85

**Figure 1:**
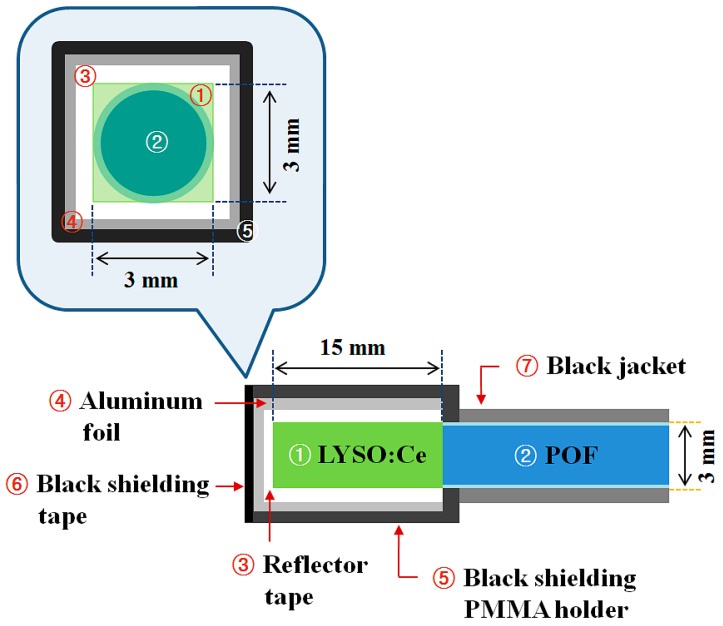
Schematic diagram of a sensing probe of a FORS.

Figure 1 shows a schematic diagram of the sensing probe of the FORS proposed in this study. The square-shaped LYSO:Ce crystal with dimensions of 3 × 3 × 15 mm^3^ was glued with optical grease (BC-630, Saint-Gobain Ceramic & Plastics, Inc., Hiram, OH, USA) to the distal end of a POF. The surface of the POF was polished with optical graded pads before coupling. The outer surface of the LYSO:Ce crystal was surrounded by polytetrafluoroethylene (PTFE, Teflon^®^, Wilmington, DE, USA) reflector tape (BC-642, Saint-Gobain Ceramic & Plastics, Inc.) to increase the scintillating light-collection efficiency. Furthermore, an aluminum foil, black shielding tape, and a black PMMA holder were used to intercept ambient light noise, as illustrated in Figure 1.

As gamma-ray emitters, two solid-disc type radioactive isotopes with the same dimension (Spectrum Techniques, LLC., Oak Ridge, TN, USA), Cs-137 and Co-60, were used. The diameter and the thickness of this solid disc-type gamma source are 25.6 mm and 2.68 mm, respectively. The physical properties of commercially available gamma-ray emitters used in this study are listed in Table 2. The photopeak (or full-energy peak) of gamma-rays generated from Cs-137 is 661.6 keV and Co-60 produces two distinct gammas, 1173 and 1332 keV [3].

**Figure 2:**
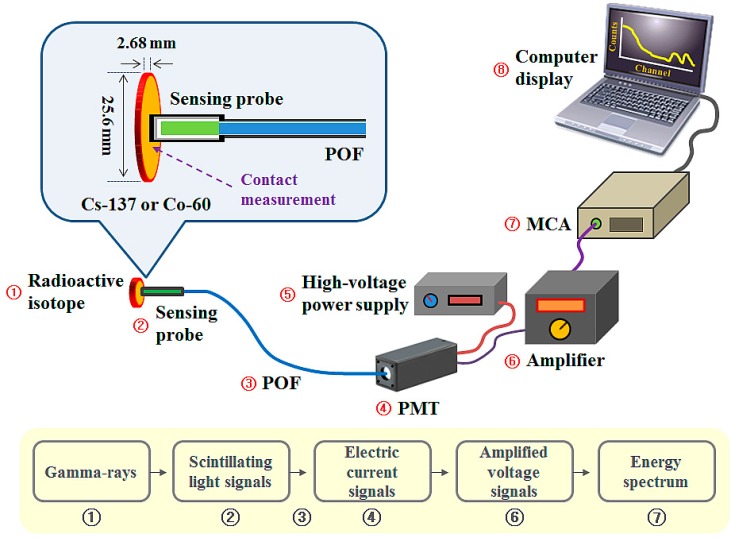
Experimental setup for measuring gamma-ray energy spectra using a FORS and gamma-ray emitters.

Figure 2 shows the experimental setup for measuring gamma-ray energy spectra using the FORS and gamma-ray emitters. A PMT-amplifier system (Hamamatsu Photonics K.K. Co., Shizuoka, Japan) was used as a light-measuring device to measure scintillating light because of its high internal gain and reasonable quantum efficiency. This photosensor system is composed of a compact side-on PMT module (H9305-03), a low-noise amplifier (C7319), and a high-voltage power supply (C7169) that can provide the PMT with driving and control voltages. The PMT equipped with an optical fiber adapter, a subminiature type A (SMA) 905 connector, has a spectral response ranging from 185 to 900 nm and a peak sensitive wavelength of 450 nm. The generated scintillating light signals due to the interactions between the gamma-rays emitted from the radioactive isotope and the LYSO:Ce crystal in the sensing probe are guided to the PMT via the POF and converted to electric current signals [1]. The current signals from the PMT are then converted to voltage signals and amplified by the amplifier. Finally, the amplified voltage signals are measured by a compact stand-alone MCA (ORTEC^®^ EASY-MCA-8k, Advanced Measurement Technology, Inc., Oak Ridge, TN, USA) with a fast conversion time of less than 2 μs and the energy spectrum is displayed and stored through emulation software (ORTEC^®^ MAESTRO^®^-32, Advanced Measurement Technology, Inc.).

**Table 2:** Physical properties of two gamma-ray emitters.

Radioactive Isotope	Half-Life (years)	Gamma Energies (keV)	Radioactivity (μCi)
Cs-137	30.1	661.6	0.23
			0.46
			0.93
			4.66
Co-60	5.27	1173	0.93
		1332	50.77

## 3. Results

### 3.1. Determination of LYSO:Ce Length for Gamma-Ray Energy Spectroscopy

In order to obtain photopeaks in the inherent energy spectrum of a radioactive isotope, the sensing volume is very important since the size of the scintillator used in a FORS is normally small. Therefore, before the experimental study on measurement of gamma-ray energy spectra using the proposed FORS, a feasibility experiment using three LYSO:Ce crystals with area of 3 × 3 mm^2^ and length of 5, 10, and 15 mm was carried out to determine the adequate length of the LYSO:Ce crystal that can accurately measure the gamma-ray energy spectrum with photopeaks. In this test, each LYSO:Ce crystal was directly connected to the faceplate window of the PMT using a black PMMA adapter without the POF. Additionally, we calculated the energy deposition of the gamma-ray emitted from Co-60 to estimate scintillating light generated in the LYSO:Ce crystal according to the length of LYSO:Ce. 

**Figure 3:**
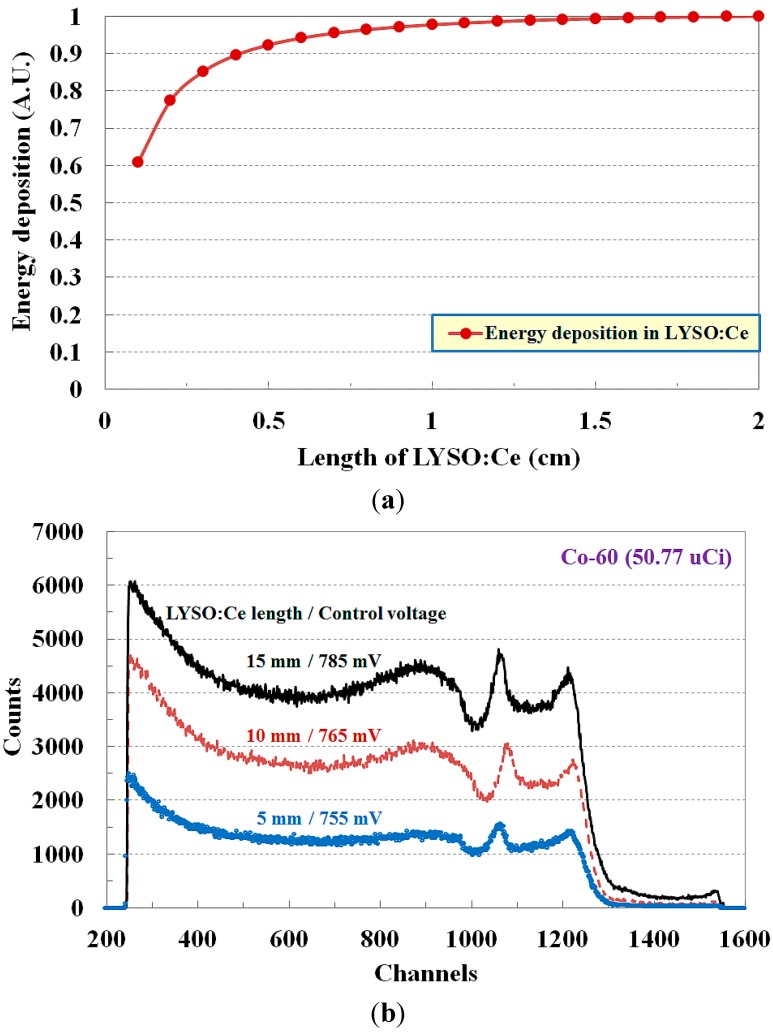
(**a**) Energy deposition of the electrons owing to the gamma-ray emitted from Co-60 according to the length of LYSO:Ce obtained by using a MCNPX simulation and (**b**) Variation of gamma-ray energy spectrum according to the length of LYSO:Ce.

Figure 3a shows the energy deposition of the electrons owing to the gamma-rays emitted from Co-60 according to the length of LYSO:Ce obtained by using Monte Carlo N-particle extended transport code (MCNPX). From the simulation results, the calculated energy deposition of the electron induced by the gamma-ray inside the LYSO:Ce crystal has a maximum value when the length of the LYSO:Ce crystal is about 15 mm. Figure 3b shows the gamma-ray energy spectra for Co-60 with a radioactivity of 50.77 μCi according to the length of the LYSO:Ce crystal. As the length of the LYSO:Ce crystal is increased from 5 to 15 mm, higher control voltages were supplied on the PMT, as shown in Figure 3b, and they were adequate to obtain inherent photopeaks of Co-60 in the gamma-ray energy spectra. Due to the fact that the absorption of primary gamma-rays and secondary charged particle energies increases as the length of the LYSO:Ce crystal is increased from 5 to 15 mm, the counts of the scintillating light generated in the LYSO:Ce crystal were also increased, and thus more light signals having energy spectrum information were transmitted to the PMT through the transparent LYSO:Ce crystal. In addition, the full width at half maximum (FWHM) of the measured photopeak was decreased as the length of the LYSO:Ce crystal became greater. As a result, the two photopeaks corresponding to photoelectrons were matched well with the inherent photopeaks of Co-60 when the length of the LYSO:Ce crystal was 15 mm. From the simulation and experiment results, it was accordingly assumed that the optimum length of the LYSO:Ce crystal is about 15 mm in our experimental setup for gamma-ray energy spectroscopy considering the absorption of charged particle energy.

### 3.2. Gamma-Ray Energy Spectroscopy Using the Proposed FORS and the Conventional HPGe Detector

**Figure 4:**
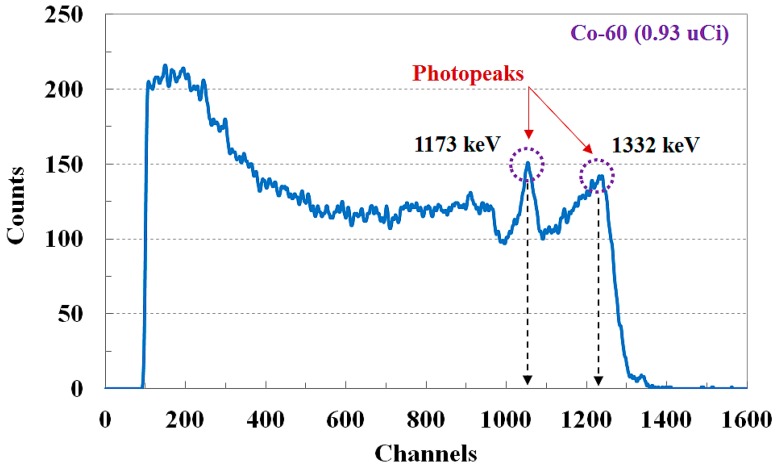
Uncalibrated gamma-ray energy spectrum for Co-60.

In this study, we developed a novel FORS using a spectroscopic technique and measured the gamma-ray energy spectra to discriminate species of radioactive isotopes. The measured gamma-ray energy spectrum for Co-60 using the FORS and the MCA at room temperature is shown in Figure 4. In this experiment, using a square-shaped LYSO:Ce crystal with dimensions of 3 × 3 × 15 mm^3^, the length of the POF and the control voltage of the PMT power-supply were fixed at 1 m and 915 mV, respectively. After a collection time of 180 s, the uncalibrated MCA spectrum was plotted using the collected data. By using the two photopeaks of Co-60, the energies that correspond to each channel were determined in order to perform calibration.

**Figure 5:**
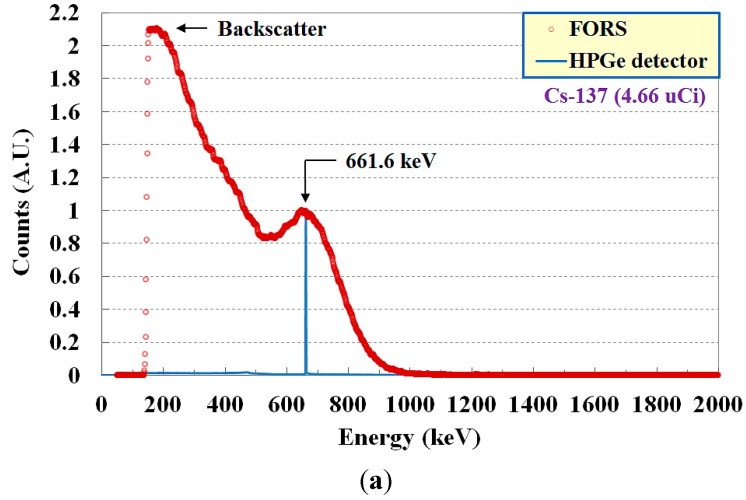

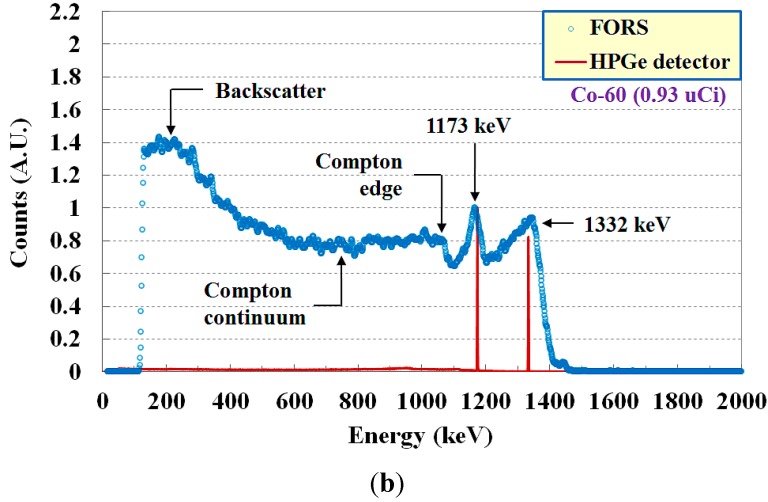
Calibrated gamma-ray energy spectra for (**a**) Cs-137 and (**b**) Co-60.

Figure 5 shows the calibrated gamma-ray energy spectra for Cs-137 with a radioactivity of 4.66 μCi and Co-60 with 0.93 μCi. The photopeaks obtained by employing a conventional HPGe spectroscopy detector (GC3018, CANBERRA Industries, Inc., Meriden, CT, USA) with a bias voltage of 2500 V, a liquid nitrogen (LN_2_) level gauge (Model 7185, CANBERRA Industries, Inc.), and a digital signal analyzer (Lynx^®^ digital MCA, CANBERRA Industries, Inc.) with Genie 2000 software were also presented in Figure 5. The photon counts measured at each energy by using two different detectors were normalized to the photopeak counts measured at 661.6 keV for Cs-137 and at 1173 keV for Co-60. Each measured photopeak was well matched with the inherent gamma-ray energy peaks of Cs-137 and Co-60 obtained by using the regularly calibrated HPGe detector, as shown in Figure 5a,b [3,19,20,21]. The backscatter peaks, which were caused by the influence of surrounding materials of the sensing probe, and a Compton edge of Co-60 were also detected. Therefore, the FORS could measure the gamma-ray emitters using energy spectroscopy and a spectral analysis (mainly photopeaks), making it possible to discriminate the species of radioactive isotopes.

### 3.3. Energy Spectrum of Mixed Radioactive Isotopes

**Figure 6:**
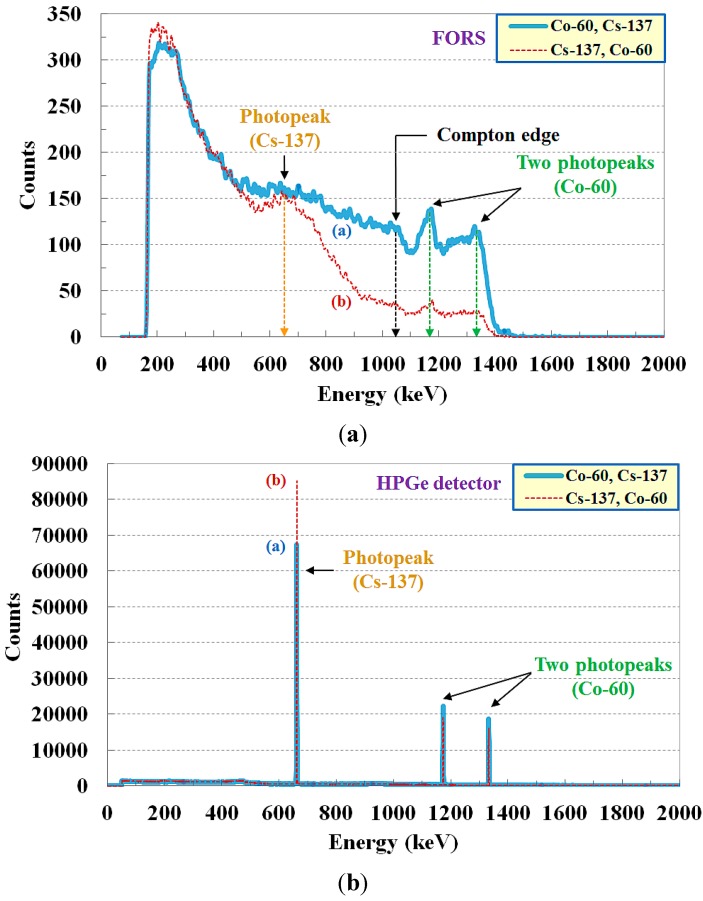
Gamma-ray energy spectra measured by using (**a**) the FORS and (**b**) the HPGe spectroscopy detector when two radioactive isotopes were mixed.

The energy spectra of gamma-rays emitted from mixed radioactive isotopes of Cs-137 and Co-60 with an almost identical radioactivity of 0.93 μCi were measured by using the FORS and the HPGe detector. In Figure 6, the solid line (a) indicates that Co-60 was located in front of Cs-137, and *vice versa* for the dotted line (b). Cs-137 emits one gamma-ray at 661.6 keV and the measured photopeak is easily distinguishable on the spectrum. On the dotted line (b), the energy peak of Cs-137 is more clearly observed than that of the solid line (a) because Cs-137 was located near the sensing probe. In the case of Co-60, two photopeaks, at 1173 and 1332 keV, were successfully observed despite that Co-60 was placed with Cs-137. In addition, the photopeak counts of Co-60 on the solid line (a), which were measured when Co-60 was located in front of Cs-137, were higher than those of the dotted line (b). The photopeaks of the gamma-ray energy spectra measured by using the FORS were well matched with those of the HPGe detector although the count rate (*i.e*., counts per unit time) of the FORS was substantially lower than that of the HPGe detector, as shown in Figure 6. The experimental results demonstrate that the proposed FORS could discriminate each gamma-ray emitter using energy spectra, even when radioactive isotopes were mixed.

### 3.4. Response of the FORS According to Radioactivity

**Figure 7:**
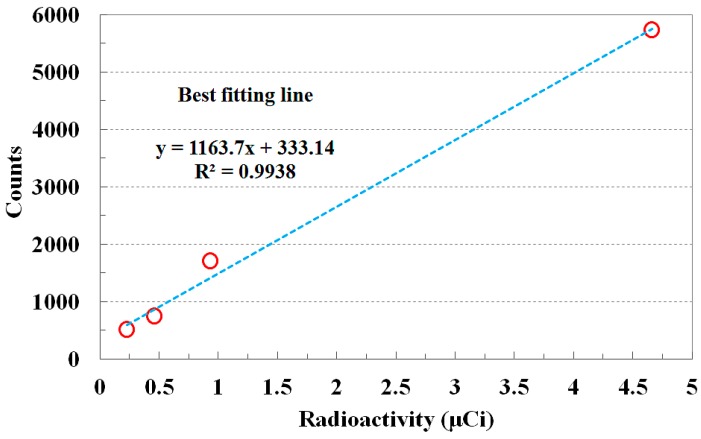
Response of the FORS according to the radioactivity variation of Cs-137.

Figure 7 shows the relationship between the radioactivities of Cs-137 and the measured photon counts. With increasing radioactivity of Cs-137, the counts of scintillating light generated from the sensing probe also increased over the same collection time. This shows that the amount of scintillating light depends on the radioactivity of a radioactive isotope. The *R*^2^ value, which is called the correlation coefficient, of a best-fitted line was found to be 0.9938. This value, which varies from 0 to 1, represents the accuracy of the correlation between the measured data and the fitted line. 

### 3.5. Variation of Energy Spectrum According to Length and Diameter of POF

**Figure 8:**
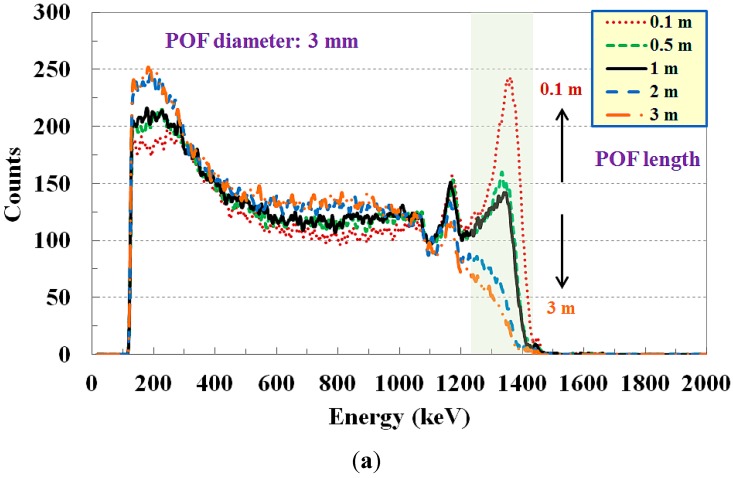

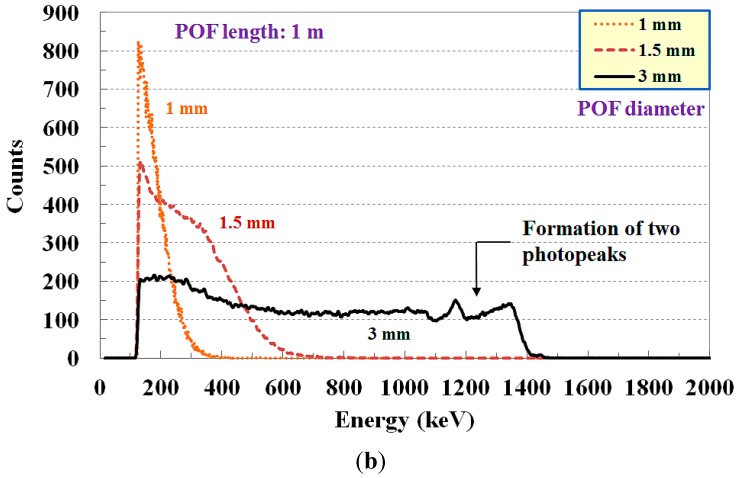
Variation of measured gamma-ray energy spectrum according to (**a**) the length and (**b**) the diameter of the POF.

Figure 8 shows the variation of the energy spectrum for Co-60 according to the length and diameter of the POF, which serves as a light guide to connect the sensing probe and the PMT. In this test, the control voltage of the PMT power supply was fixed at 915 mV, the optimum voltage to obtain typical Co-60 spectroscopy results for a POF with a length of 1 m and a diameter of 3 mm. 

Increasing the length of the POF from 0.1 to 3 m, the original wavelength of the scintillating light might be transmitted differently according to the optical properties of the step-index multimode POF, such as the spectral transmissivity and the material absorption. Furthermore, the FWHM of the photopeak increases as the length of the POF is increased because of a modal dispersion, as shown in Figure 8a. Increasing the diameter of the POF from 1 to 3 mm, more propagation modes are permitted in the POF and more light signals having energy spectrum information are transmitted to the PMT via the POF; these effects enable the formation of the photopeaks in spite of the pulse broadening due to the increment of the modal dispersion, as shown in Figure 8b. Meanwhile, the distortions due to the length and diameter of the POF can be corrected to some extent by changing the control voltage of the PMT power supply in a permitted range.

## 4. Discussion

Many kinds of radiation sources produce gamma-rays and often contaminate environments. Therefore, it is important to have an accurate method to determine the species of radionuclide that emits the gamma-rays. The conventionally-applied method for detecting and discriminating gamma-rays in nuclear and medical facilities is a conventional gamma-ray spectroscopy technique using fixed-type radiation detection systems including NaI:Tl and HPGe detectors. This method should be performed on site or at a laboratory, as it is necessary to take and transport the radioactive sample. Therefore, it is impossible to perform gamma-ray energy spectroscopy for the radioactive isotopes located in the inaccessible places due to the large sensing volume of the NaI:Tl and HPGe detectors. Furthermore, there is a critical disadvantage in that human radiation exposure is inevitable when the radioactivity of the gamma emitter is quite high. In order to overcome these problems, many different types of FORSs have been investigated and developed by many research teams. However, most FORSs can only measure the light intensity of scintillation signals or the total counts of the energy spectrum; therefore, they have not been used for accurate discrimination of the radioactive isotopes.

The FORS system proposed by our research team is composed of a small LYSO:Ce crystal with dimensions of 3 × 3 × 15 mm^3^, a 1 m-long POF with a diameter of 3 mm, a PMT-amplifier system with a control voltage of 915 mV, and an MCA. In this FORS system, the compositions and each parameter were optimized to measure the energy spectra of the gamma-rays emitted from Cs-137 and Co-60 with minimized dead time. Therefore, if the energy of a radioactive isotope is changed, the species or the dimensions of both the scintillator and the POF and the control voltage of the PMT-amplifier system also should be changed because a small sized scintillator in the sensing probe of the FORS cannot completely absorb high-energy radiation and the transmission or attenuation rate of the scintillating light with low intensity is dependent on the physical and optical properties of the scintillator and the POF.

To compare the gamma-ray energy spectrum measured by the FORS, unfortunately, we used a Ge semiconductor diode-based HPGe detector (*i.e.*, standard electrode coaxial Ge detector) instead of a scintillation detector based on an NaI:Tl crystal. Accordingly, it was only possible to compare each photopeak measured by using the FORS and the HPGe detector. From the experimental results, the photopeaks of the gamma-ray energy spectra measured by using the FORS were well matched with those of the HPGe detector; however, the FWHM related to the energy resolution was much larger in the FORS compared to the HPGe detector. From the viewpoint of energy resolution, a scintillator detector offers relatively low resolution as a radionuclide identifier (RID) compared to a semiconductor detector [22]. However, the diameter and the length of the coaxial HPGe detector used in this study are 76 mm and 110 mm, respectively, and hence its sensing volume is much larger than that of the FORS. Furthermore, the HPGe detector must be cooled to avoid excessive thermally-generated leakage current while the proposed FORS can measure energy spectra at room temperature without cooling.

## 5. Conclusions

In conclusion, the proposed FORS can be used to measure the counts of scintillating light according to the radioactivity relevant to radioactive contamination and to discriminate species of radioactive isotopes by measuring their inherent energy spectra, even when gamma-ray emitters are mixed. In hazardous nuclear and medical environments, the FORS offers many advantages over conventional radiation sensors, such as small sensing volume, adequate flexible and insertable characteristics, remote operation, *in situ* measurement, and immunity to EMI. Further studies will be carried out to fabricate and optimize a FORS with longer length POF and other inorganic scintillators for measuring energy spectra remotely. Based on the results of this study, it is anticipated that a novel FORS can be developed to accurately measure gamma-ray energy spectra.
